# Therapeutic potential to target sialylation and SIGLECs in neurodegenerative and psychiatric diseases

**DOI:** 10.3389/fneur.2024.1330874

**Published:** 2024-03-11

**Authors:** Jannis Wißfeld, Tawfik Abou Assale, German Cuevas-Rios, Huan Liao, Harald Neumann

**Affiliations:** ^1^Institute of Reconstructive Neurobiology, Medical Faculty and University Hospital Bonn, University of Bonn, Bonn, Germany; ^2^Florey Institute of Neuroscience and Mental Health, Faculty of Medicine, Dentistry and Health Sciences, University of Melbourne, Parkville, VIC, Australia

**Keywords:** SIGLEC, sialylation, sialic acid, microglia, neuroinflammation, neurodegeneration, Alzheimer’s disease

## Abstract

Sialic acids, commonly found as the terminal carbohydrate on the glycocalyx of mammalian cells, are pivotal checkpoint inhibitors of the innate immune system, particularly within the central nervous system (CNS). Sialic acid-binding immunoglobulin-like lectins (SIGLECs) expressed on microglia are key players in maintaining microglial homeostasis by recognizing intact sialylation. The finely balanced sialic acid-SIGLEC system ensures the prevention of excessive and detrimental immune responses in the CNS. However, loss of sialylation and SIGLEC receptor dysfunctions contribute to several chronic CNS diseases. Genetic variants of *SIGLEC3*/*CD33*, *SIGLEC11*, and *SIGLEC14* have been associated with neurodegenerative diseases such as Alzheimer’s disease, while sialyltransferase *ST8SIA2* and *SIGLEC4*/*MAG* have been linked to psychiatric diseases such as schizophrenia, bipolar disorders, and autism spectrum disorders. Consequently, immune-modulatory functions of polysialic acids and SIGLEC binding antibodies have been exploited experimentally in animal models of Alzheimer’s disease and inflammation-induced CNS tissue damage, including retinal damage. While the potential of these therapeutic approaches is evident, only a few therapies to target either sialylation or SIGLEC receptors have been tested in patient clinical trials. Here, we provide an overview of the critical role played by the sialic acid-SIGLEC axis in shaping microglial activation and function within the context of neurodegeneration and synaptopathies and discuss the current landscape of therapies that target sialylation or SIGLECs.

## 1 Introduction

Neurodegenerative diseases are characterized by the gradual and chronic loss of neuronal function and cells, resulting in cognitive and physical impairment that eventually leads to the patient’s death. Among the most prevalent neurodegenerative diseases in industrialized countries are Alzheimer’s disease (AD) and Parkinson’s disease (PD) (1). The hallmarks of these diseases are the deposition of protein aggregates within neurons or the extracellular space, coupled with pronounced dysfunction of microglia, the resident innate immune cells of the brain. If microglia fail to clear the extracellular deposits of protein aggregates, they progressively adopt an inflammatory profile. While the protein aggregates themselves can be toxic to neurons (2–4), they can also trigger an aberrant and detrimental activation of microglia (5, 6). Such aberrantly activated or reactive microglia subsequently produce reactive oxygen species and pose a toxic threat to synapses and neurons, thus exacerbating the disease (7). In physiological conditions, a microglial response restricted in time and space is important to clear cellular debris and protein aggregates. However, once the damaged tissue is cleared, microglia must transit back to a non-inflammatory phenotype to keep the central nervous system (CNS) in homeostasis. Therefore, microglia possess specific sialic acid-binding immunoglobulin-like lectin (SIGLEC) receptors that recognize intact and healthy tissue, initiating the downregulation of microglial inflammatory processes and facilitating the resolution of inflammation. These SIGLEC receptors recognize sialylated structures of the glycocalyx on host cell surface proteins and lipids. A key element of these structures is the terminal sialic acid (Sia) residue that acts as a beacon for this regulatory process. Thus, SIGLEC receptors on microglia detect sialylation on intact host cells and the receptor engagement subsequently antagonizes inflammatory activatory signaling pathways. This mechanism ensures a continuous control of microglial actions against host cells. However, this system of sensing self-structures also allows for an immediate response to invading pathogens lacking this specific sialylation flag.

Within this review, we will summarize the importance of the Sia-SIGLEC axis in the context of microglial activation and function and its role in the development of neurodegenerative and psychiatric diseases. Furthermore, we will discuss current and potential SIGLEC- or sialylation-targeting therapies for the treatment of neurodegenerative diseases.

## 2 Main text

### 2.1 Sialylation—a checkpoint inhibitor for the innate immune system protecting the CNS

**Figure 1:**
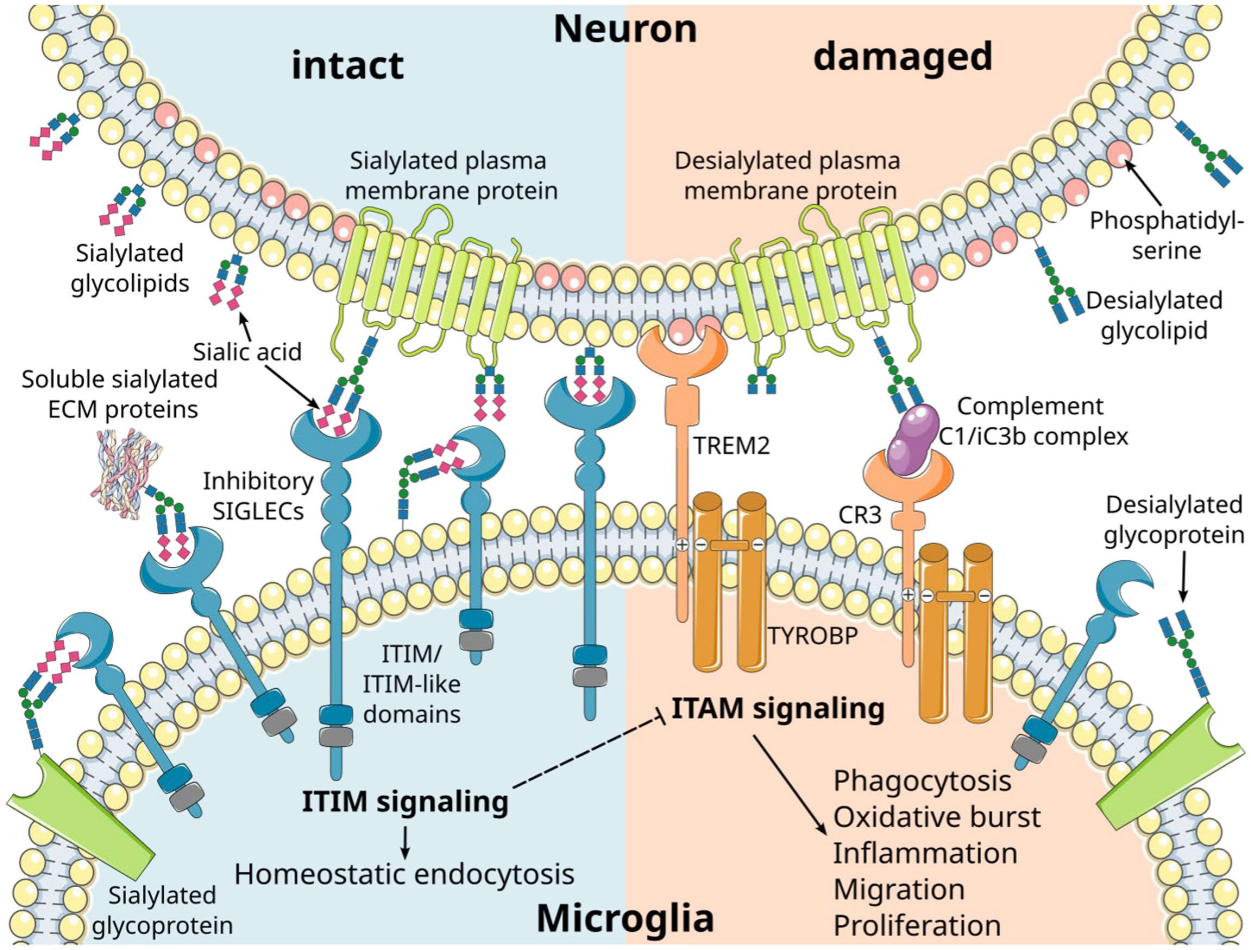
Inhibitory microglial SIGLEC receptors recognize sialylated ligands and inhibit microglial responses. Sialic acids are typically found on the terminal position of sialylated glycolipids and glycoproteins on mammalian cells. Recognition of the sialylated ligands by immunoreceptor tyrosine-based inhibition motif (ITIM)-signaling SIGLEC receptors leads to inhibition of intracellular signals emanating from immunoreceptor tyrosine-based activation motif (ITAM)-signaling receptors. Consequently, the ITIM signaling pathway exerts a regulatory influence over various microglial responses, including phagocytosis, oxidative burst, inflammation, migration, and proliferation. SIGLEC receptors can take up their ligands via the ITIM signaling pathway, leading to homeostatic endocytosis. This dual functionality allows them to maintain cellular balance. Several cell membrane receptors likely converge on the ITAM/ITIM response to generate distinct signals through various intracellular second messenger pathways. Phosphatidylserine residues exposed on the outer membrane leaflet of damaged cells are recognized by triggering receptor expressed on myeloid cells 2 (TREM2), resulting in activatory signaling via the associated ITAM-containing protein transmembrane adapter protein transmembrane immune signaling adaptor TYROBP. Moreover, desialylation serves as a trigger for complement opsonization of factor C1 and complement activation via the C3 convertase. CR3, complement receptor 3 (depicted as a heterodimer of CD11b/lTGAM and CD18/ITGB2); ECM, extracellular matrix. Parts of the figure were drawn by using original or modified pictures from Servier Medical Art. Servier Medical Art by Servier is licensed under a Creative Commons Attribution 3.0 Unported License (https://creativecommons.org/licenses/by/3.0/).

The glycocalyx is a complex and dynamic structure that covers the outer surface of nearly all types of cells. It is composed of a diverse array of carbohydrates, which are attached to underlying proteins and lipids forming the plasma membrane (Figure 1). The glycocalyx plays a pivotal role in many biological processes including cell recognition, cell adhesion, and signal transduction (8, 9). Sialic acids, also called neuraminic acids, typically are the terminal carbohydrates of the glycocalyx of mammalian cells. They comprise a family of monosaccharides with a nine-carbon backbone. The sialylation on the cell surface serves structural, biophysical, and receptor-binding functions. Sialic acids are highly negatively charged and form a hydrophilic hydration shell. This shell increases the dynamic volume of the molecules they are attached to, effectively preventing non-specific interactions between cells. Consequently, stem cells use polysialylated molecules on their cell surface to facilitate motility and promote plasticity. In addition, the highly negatively charged sialylated glycocalyx can mask underlying cell membrane molecules, which only become visible to receptors of other cells after undergoing neuraminidase-mediated desialylation (10). This neuraminidase-mediated removal of the sialylation cap is essential for unveiling the latent status of immune cells, enabling both inflammatory responses as well as phagocytic activities. For instance, in cases of tissue damage, the sialylation cap on tissue macrophages is removed, allowing activation, toll-like receptor (TLR) signaling and the subsequent phagocytic removal of their targets (11).

The sialylated glycoproteins and glycolipids of the glycocalyx are ligands for several receptors, most of which belong to the SIGLEC receptor family and the complement cascade (Figure 1). These specific interactions are based on the binding of the ligands to the sialic acid moiety, along with the underlying carbohydrate chain and its linkage. Furthermore, these receptors exhibit specificity for the precise subtype of the sialic acid, namely N-acetylneuraminic acid (Neu5Ac) or N-glycolylneuraminic acid (Neu5Gc) that are found in mammalian cells, as well as their O-acetylated derivatives (10). Interestingly, humans have lost the ability to synthesize Neu5Gc due to a lineage-specific loss-of-function deletion in the gene encoding the enzyme cytidine monophosphate-N-acetylneuraminic acid hydroxylase (CMAH) (12). As a result, Neu5Ac is the only subtype of sialic acid produced by human cells. However, Neu5Gc from dietary sources can be metabolically incorporated into human tissues and may function as a xeno-antigen. Antibodies against Neu5Gc-containing epitopes are frequently detected in humans, with their levels and repertoire being associated with dietary intake of red meat and dairy products (13–17). Additionally, it is possible that a high oxidative burst of cells can modify Neu5Ac to Neu5Gc (18). The exact pathophysiology of Neu5Gc incorporation into human tissues and Neu5Gc-specific antibody formation remains unclear. However, it has been proposed that this phenomenon may exacerbate cancer (15, 16) and contribute to cardiovascular diseases in mice (19).

The sialic acids for the sialylated glycans of the glycocalyx are synthesized in the cytoplasm and subsequently attached to the underlying glycans in the Golgi apparatus by sialyltransferases that differ in their substrate specificity and the types of linkages they produce (20–22). The majority of these linkages are in an alpha configuration. Their definition is based on the carbon of the acceptor glycan to which the anomeric carbon of the transferred sialic acid (carbon 2) is connected, typically resulting in α2,3 or α2,6 linkages (23, 24). Polysialic acid (polySia) is a linear homo-polymer with an α2,8 linkage found in mammals. It exhibits variable degrees of polymerization (DP), typically ranging from eight up to approximately 30 sugar residues. In the CNS, polySia can extend to up to 400 sugar residues on neural cell adhesion molecules (NCAM) (25). Within the CNS, most sialic acids are found on glycolipids, namely gangliosides, while polySia is primarily detected on glycoproteins, such as NCAM, cell adhesion molecule 1/synaptic cell adhesion molecule 1 (CADM1/SynCAM1), neuropilin 2 (NRP2), and Golgi glycoprotein 1/E-selectin ligand-1 (GLG1/ESL-1) (26–30). PolySia expression has been described in various contexts, including developing neurons and glial cells, adult neural stem cells, migrating neuroblasts in the two zones with adult neurogenesis, synapses across all brain regions, and regions with synaptic plasticity (31). Apparently, the highly negatively charged polySia plays a pivotal role in supporting neuronal outgrowth, regeneration, and synaptic plasticity, thereby facilitating motility and plasticity. However, these functions are also exploited by glioblastomas to promote cancer growth, migration, and metastasis formation, in which polySia-NCAM overexpression is associated with poorer disease-free and overall survival (32, 33).

The function of the sialylation of glycolipids and glycoproteins has been studied in mice by gene deletion of the enzyme glucosamine (UDP-N-acetyl)-2-epimerase/N-acetylmannosamine kinase (*Gne*), which is essential for the cellular synthesis of sialic acid (34). While homozygous *Gne*-deficient mice (GNE^−/−^) with complete loss of sialic acid synthesis show lethality during embryonic development (34), slightly reduced sialylation in heterozygous GNE^+/−^ middle-aged mice results in a complement C3-mediated loss of neurons (35). Neurites with an intact sialylated glycocalyx are protected, while desialylated neurites are cleared by microglia in a complement C1 binding and complement receptor 3 (CR3)-mediated process (36) (Figure 1). The anti-inflammatory effects of sialylation on the complement system are also mediated by inhibitory complement factors and the inactivation of activatory complement factors. For instance, complement factor H binds to α2,3-linked sialic acids on the cell surface and is known to bind the opsonin C3b, thereby inhibiting the formation and inducing the disassembly of the alternative C3-convertase (37–39). Furthermore, low molecular weight polysialic acid has been shown to have the capacity to sequester the positively charged complement protein properdin (40). Experimentally, it prevented activation of the alternative complement pathway and protected susceptible murine hepatoma cells and rat neuroblastoma cells from complement-mediated cell death *in vitro* (40). As previously discussed, the anti-inflammatory effects of sialylation on microglia and invading immune cells are predominantly mediated through their inhibitory SIGLEC receptors. In THP-1 macrophages, low molecular weight polysialic acid decreased the gene transcription of inflammatory mediators, which were induced by lipopolysaccharide (LPS) in a SIGLEC-11-mediated mode of action (41). Furthermore, polysialic acid also interfered with pro-inflammatory effector molecules of neutrophils. Polysialic acid and nanoparticles coupled with α2,8-linked oligosialic acid chains prevented the formation of neutrophil extracellular traps (NETs) and reduced the production of reactive oxygen species by decreasing the cytotoxic activity of histones (42, 43).

While mice with one mutant allele of the *Gne* gene exhibit a brain phenotype, humans with genetic mutations in *GNE* show muscle-related disorders. Patients diagnosed with GNE myopathy display impaired or insufficient sialylation in their muscles, which has been associated with local inflammation and oxidative damage. Interestingly, the muscle phenotype has also been associated with local aggregation of proteins like amyloid-β and phosphorylated tau, thus resembling the inflammatory aggregation phenotype of neurodegenerative diseases in muscle tissue (44, 45). Sialylation also plays a role in AD. Studies on cerebrospinal fluid (46), serum (47), and postmortem brain tissue (48) of AD patients have revealed decreased protein sialylation and a reduction in enzymes responsible for protein sialylation. Loss of the sialic acid cap or protein desialylation is considered a molecular indicator of protein aging that triggers protein turnover (49–52). Conversely, the removal of sialic acid residues from proteins and lipids in the lysosome appears to be essential for a proper degradation of the amyloid protein precursor (APP) (53) and consequently preventing the accumulation of amyloid-β in cells. Deficiency of the sialic acid-cleaving enzymes neuraminidase-3 (*Neu3*) and *Neu4* in mice resulted in the accumulation of undigested ganglioside GM3 in lysosomes of microglia, vascular pericytes, and neurons (54). Interestingly, neuraminidases either have very limited efficacy or fail to cleave α2,8-linked Neu5Gc (55). Therefore theoretically, polysialic acid, which is enriched in the central nervous system, might not be properly digested within the lysosomes if Neu5Gc is incorporated. This might be a possible explanation for the puzzling finding that the CNS, under normal conditions, is almost devoid of Neu5Gc in all species (56).

Thus, sialylation serves as a checkpoint for the innate immune system to prevent detrimental immune responses against host cells and cellular structures in the brain, including synapses and neurons. However, it is crucial that sialic acid residues are removed in the lysosome by specific enzymes to ensure proper digestion and recycling.

### 2.2 Inhibitory SIGLECs are expressed on microglia to sense sialylation and prevent overt oxidative damage

**Figure 2:**
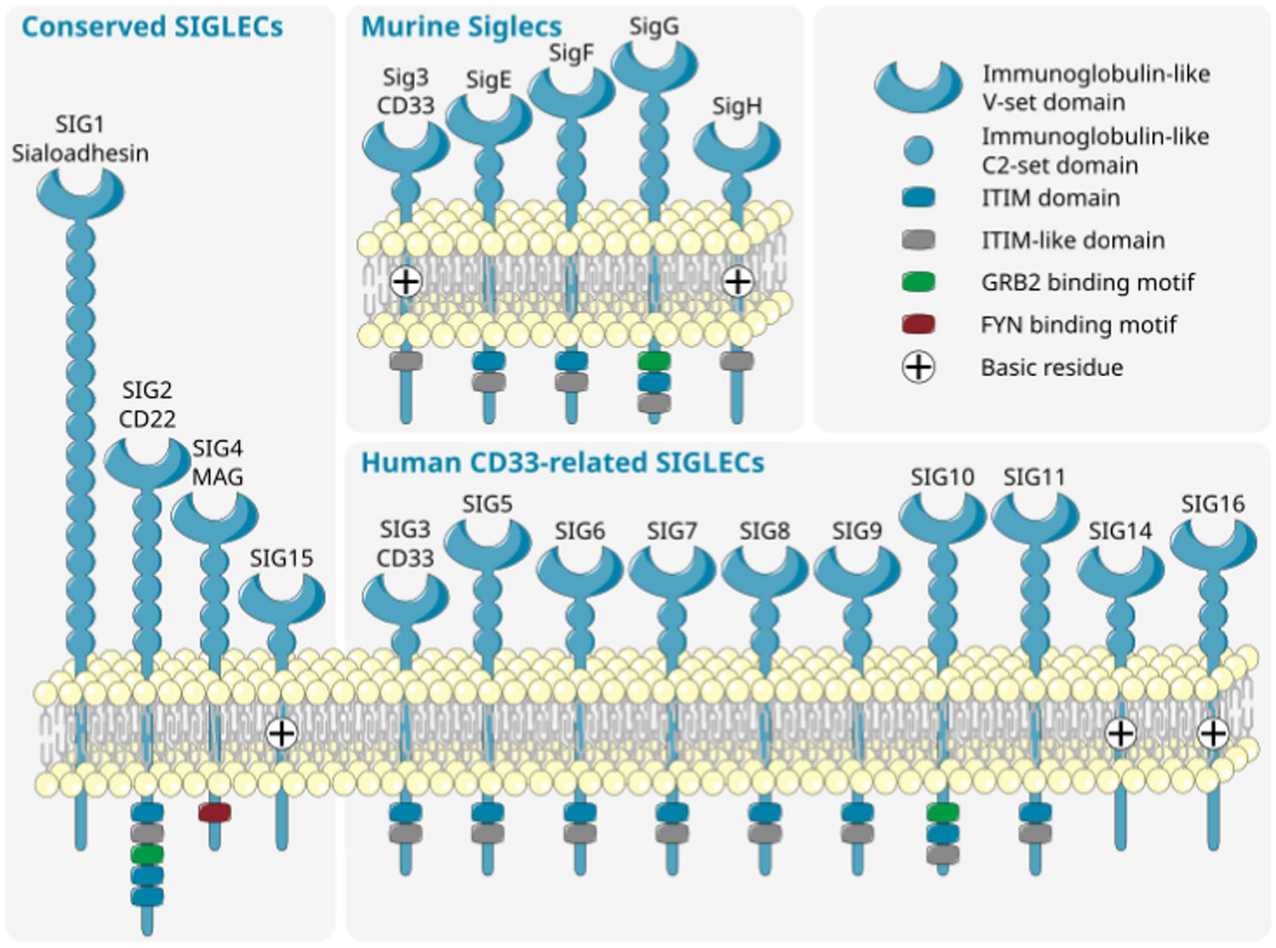
Diversity of murine (top right) and human (bottom right) CD33-related SIGLECs together with conserved SIGLECs (left). SIGLECs are type 1 membrane proteins with an N-terminal variable sialic acid recognition domain (V-set Ig-like), a variable number of constant C2-set Ig-like domains, and with few exceptions intracellular signaling motifs. The CD33-related SIGLECs substantially differ in composition, ligand recognition, and intracellular signaling motif between distinct species. ITIM, immunoreceptor tyrosine-based inhibition motif; SIG/SIGLEC, Sialic acid-binding immunoglobulin-like lectins; GRB, growth factor receptor bound protein; FYN, FYN proto-oncogene, Src family tyrosine kinase. Parts of the figure were drawn by using original or modified pictures from Servier Medical Art. Servier Medical Art by Servier is licensed under a Creative Commons Attribution 3.0 Unported License (https://creativecommons.org/licenses/by/3.0/).

**Figure 3:**
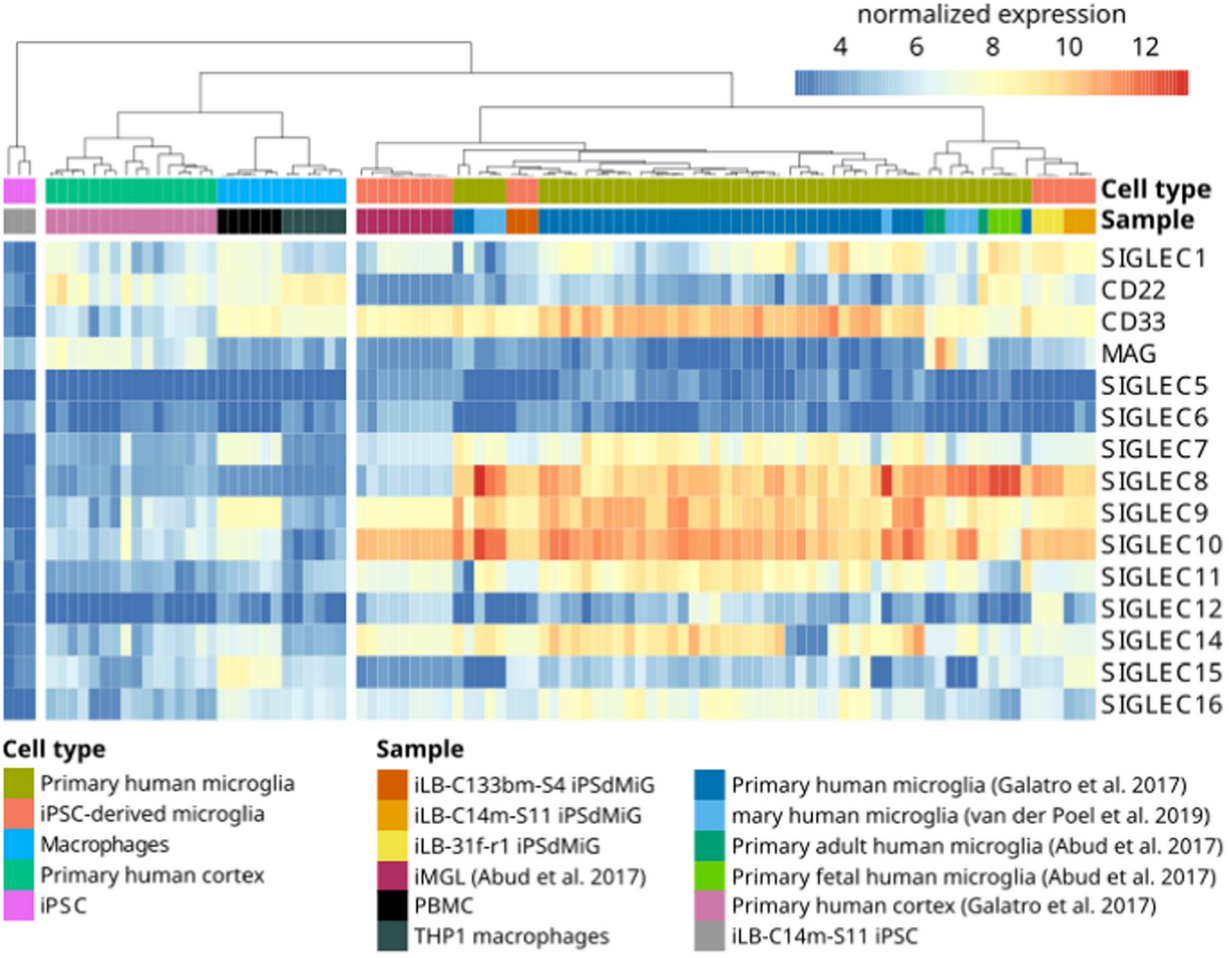
Gene transcripts of SIGLEC receptors are detected by RNAseq in human microglia. Heatmap of RNAseq data from human iPSC (iLB-C14m-S11; in-house produced), human iPSC-derived microglia (iLB-C133bm-S4, iLB-31f-r1, iLB-C14m-S11; in-house produced), human iPSC-derived microglia-like cells (61), fetal and adult human microglia (61), primary human microglia (60, 62), human cortex (60), human peripheral blood mononuclear cells (PBMCs; in-house produced) and human THP1 macrophages (in-house produced). Microglia show evidence for *SIGLEC1*, -*3*, -*7*, -*8*, -*9*, -*10*, -*11*, -*14*, and -*16* gene transcripts. CD22 = SIGLEC-2; CD33 = SIGLEC-3; MAG = SIGLEC-4; iPSC, induced pluripotent stem cells; iMGL, iPSC-derived microglia-like cells, iPSdMiG, iPSC-derived microglia.

The sialylation status of the cellular glycocalyx is predominantly sensed by SIGLEC receptors. SIGLECs are type-I lectins mainly expressed on innate immune cells. They can be broadly divided into two subgroups based on sequence similarity and evolutionary conservation: (i) evolutionary-conserved SIGLECs and (ii) the rapidly evolving CD33-related SIGLECs (Figure 2). The conserved SIGLECs include sialoadhesin (SIGLEC-1), CD22 (SIGLEC-2), myelin-associated glycoprotein (MAG; SIGLEC-4), and SIGLEC-15. The remaining CD33-related (CD33r) SIGLECs in humans are CD33 (SIGLEC-3), SIGLEC-5 to -11, -14, and *-*16, and in mice CD33 (Siglec-3) and Siglecs-E to -H (57–59). Through a meta-analysis using publicly available (60–62) as weel as in-house generated microglia and macrophage mRNA sequencing datasets, we observed that human primary microglia show gene transcripts of *SIGLEC1, -3* (*CD33*), *-7* to *-*11, *-14*, and *-*16, while in both macrophage cell types (PBMC-derived and THP1) only *SIGLEC1*, *-2*, and *-3* (*CD33*) were consistently detected. However, gene transcripts of *SIGLEC7*, *-9*, *-10*, *-14*, and *-15* were also detected at low levels in PBMC-derived macrophages (Figure 3). Furthermore, currently available techniques to generate microglia from human induced pluripotent stem cells (iPSCs) demonstrated comparable levels of SIGLEC expression to their primary counterparts (63) (Figure 3). Thus, human microglia demonstrate the expression of various SIGLEC receptors. In line, expression of Siglec-3/CD33 and Siglec-E to -H, next to evolutionary conserved SIGLECs, has been described in mouse microglia (64–69).

SIGLECs function by recognizing sialylated molecules through their extracellular N-terminal domain, while the C-terminal cytoplasmic tail carries immunoreceptor tyrosine-based inhibitory motifs (ITIMs) or, less frequently, a basic residue that recruits immunoreceptor tyrosine-based activatory motif (ITAM) signaling molecules such as transmembrane immune signaling adaptor TYROBP (TYROBP)/DNAX-activation protein 12 (DAP12; Figure 1) (59, 70–73). Despite SIGLECs’ shared preference for sialic acid-containing glycans, each SIGLEC has a unique target binding profile (57). SIGLECs bind in *cis* to sialylated membrane molecules expressed on their own cell surface (74), but can also engage in *trans* interactions with high-affinity ligands that typically outcompete *cis* ligands for binding (75). These *trans* interactions enable microglia to sense the intact glycocalyx on neighboring cells, thereby maintaining microglial homeostasis (Figure 1). SIGLEC-ITIM signaling can inhibit microglial activation, inflammation, phagocytosis, and oxidative burst, and thus, acts as a safety mechanism to protect healthy host cells from damage. The majority of SIGLECs, including human SIGLECs-1 to -5, -7 to -11, and mouse Siglec-E to -H, also act as endocytic receptors facilitating the uptake of small cargos from the cell surface to endosomes (57). These SIGLEC-mediated endocytic functions may contribute to the homeostatic turnover of sialylated glycoproteins and glycolipids.

In mice, Siglec-E is one of the major inhibitory Siglecs found on microglia. Loss of Siglec-E on mouse microglia resulted in a very strong oxidative burst when challenged with neuronal cell debris (65). *Siglece*-deficient mice showed oxidative damage to cellular DNA, proteins, and lipids in all organs. This damage was attributed to an imbalanced reactive oxygen species (ROS) metabolism and a secondary impairment in the detoxification of reactive molecules. Consequently, *Siglece*^−/−^ mice showed signs of accelerated aging, displayed behavioral abnormalities, and had a reduced life span (76). Interestingly, a strong positive correlation between the lifespan and the number of inhibitory signaling SIGLEC genes was observed when analyzing 26 species. This suggests that the careful regulation of reactive oxygen species through inhibitory SIGLEC receptors appears to play a crucial role in promoting healthy aging (77). Moreover, Li and colleagues observed that systemic LPS treatment in mice led to a dose-dependent induction of *Siglece* in the brain. *Siglece*-deficient mice revealed an exacerbated hippocampal microgliosis following LPS treatment and increased neuronal cell death in oxygen–glucose deprivation (OGD)-treated cortical cultures. Subsequent to middle cerebral artery occlusion (MCAO), *Siglece* was substantially upregulated in brain tissues, and *Siglece* knockout mice exhibited enhanced neurological deficits and larger infarcts (78). Likewise, conditional deletion of *Siglece* in microglia also led to increased production of pro-inflammatory cytokines and upregulation of phagocytosis in a glioblastoma animal model (79). These findings underscore the crucial anti-inflammatory and neuroprotective roles of Siglec-E in several brain disease models.

Together, inhibitory SIGLECs play a crucial role in maintaining microglial homeostasis and preventing oxidative damage through sensing the sialylated glycocalyx on neighboring host cells.

### 2.3 Activatory microglia pathways that trigger neurodegeneration are antagonized by inhibitory SIGLEC signaling

Inhibitory SIGLEC receptor signaling antagonizes activatory signals emanating from complement receptor 3 (CR3) and triggering receptor expressed on myeloid cells 2 (TREM2) receptors via the ITAM-containing TYROBP/DAP12 (Figure 1). This counteraction is initiated through the phosphorylation of SIGLECs’ intracellular ITIMs by Src family kinases after ligand binding. Subsequently, the tyrosine-phosphorylated ITIMs recruit tyrosine phosphatases, such as Src homology region 2 domain-containing phosphatase 1 (SHP-1/PTPN6) or 2 (SHP-2/PTPN11), which can dephosphorylate signaling molecules along the ITAM signaling cascade including ITAMs itself (73, 80, 81). The activation of microglial SIGLEC-11 by polysialic acid on NCAM has demonstrated a neuroprotective effect in a murine neuron–microglia co-culture model. It suppressed proinflammatory mediators such as interleukin 1β and nitric oxide synthase 2 induced by LPS (82). Multiple molecules within the ITIM/ITAM signaling axis have been implicated in neurodegenerative diseases. For instance, genome-wide association studies have linked polymorphisms in *TYROBP*, *TREM2*, *SIGLEC3* (*CD33*), and *SIGLEC11* to AD (83–88). In addition, increased TYROBP gene transcript levels and TYROBP signaling have been observed in AD patients and AD mouse models (89, 90). Zhang et al. identified a TYROBP-driven co-expression module in human AD brain samples that correlated with complement activation (89). Furthermore, the loss of TYROBP repressed the transition from homeostatic to disease-associated microglia (DAM), including the downregulation of TREM2 and complement components in an Alzheimer’s disease mouse model (APP/PSEN1 mice). This reduction in the clinical phenotype occurred without alteration of amyloid-β burden (91). In line, TREM2 deficiency in mice was shown to have neuroprotective properties, reducing age-related inflammatory changes, accumulation of oxidized lipids, and loss of neuronal structures (92). Conversely, TREM2-triggered apolipoprotein E (APOE) signaling was associated with a shift toward a neurodegenerative microglial phenotype, characterized by a loss of the ability to maintain brain homeostasis (93). Furthermore, complement activation in the brain has been known to contribute to early synapse loss in AD (94). Experimentally, blood-derived fibrinogen activated microglial CD11b/CD18 (CR3), leading to oxidative damage and neurodegeneration in animal models of multiple sclerosis (95) and Alzheimer’s disease (96). On the other hand, DAMs, as a distinct microglia phenotype, showed an upregulation of the ITAM-signaling molecule TYROBP while concurrently downregulating microglial checkpoint genes including CX3CR1, as identified by single-cell RNA-seq in Alzheimer’s disease mouse models (97). The full activation of DAMs was shown to depend on TREM2 signaling. These highly phagocytic microglia were typically located near amyloid-β plaques in 5xFAD mice, a mouse model for Alzheimer’s disease, and in human post-mortem AD brains. Moreover, therapeutic TREM2 activation resulted in decreased amyloid-β deposition and improved clinical outcomes in 5xFAD mice (97–99).

Hence, microglial TREM2 and ITAM signaling seem to play a crucial role in clearing amyloid-beta plaques in animal models of Alzheimer’s disease (98, 100, 101). However, it is essential to carefully regulate this microglial activation in time and space, which is realized by microglial SIGLEC-ITIM signaling, to prevent excessive collateral damage, thereby mitigating neurodegeneration.

### 2.4 Involvement of sialylation and SIGLECs in neurological diseases

The CD33-related inhibitory SIGLEC receptors on microglia inhibit inflammation, phagocytosis, and the associated oxidative burst (Figure 1). Genetic variants of several SIGLEC genes have been associated with either an increased risk of developing AD or protection against it (see Table 1 for an overview of the association of sialylation and SIGLEC gene loci with neurological and neurodegenerative diseases). Carriers of the full-length form of *SIGLEC3/CD33* (*CD33M*) exhibit an increased risk of developing AD. In contrast, individuals carrying the sialic acid binding domain-deleted isoform *CD33^ΔE2^* (*CD33m*) have a reduced risk of developing AD with an average odds ratio of 0.89 (83, 85, 86). Another polymorphism in *SIGLEC3/CD33* (rs201074739), occurring at a minor allele frequency of approximately 2.4% in the European population, results in a premature termination codon due to a 4-bp deletion (112). Interestingly, this complete loss of *SIGLEC3/CD33* (rs201074739) is not significantly associated with an increased risk of developing AD (113). Therefore, the precise functional impact of the sialic acid binding domain-deleted isoform *CD33^ΔE2^* (*CD33m*) in conferring AD protection remains unclear. Of note, the *CD33^ΔE2^* variant has been shown to be primarily located in peroxisomes rather than on the cell surface (114, 115), resulting in reduced SIGLEC-3/CD33 cell surface expression on microglia. Importantly, the proportion of SIGLEC-3^+^/CD33^+^ microglia in the brain has been shown to positively correlate with the amount of amyloid-β plaques and AD progression (67, 116). Furthermore, CD33^ΔE2^-expressing microglia showed increased ITAM signaling, phagocytosis, and cytokine mRNA levels without a concurrent rise in ROS production (117, 118). These findings suggest that the CD33^ΔE2^ may confer protection against AD, either through a gain-of-function mechanism (e.g., compensatory up-regulation of other protective/inhibitory ITIM-signaling microglial receptors) or by a partial loss-of-function that indirectly influences or enhances microglial ITAM signaling and amyloid-β plaques phagocytosis, without exacerbating detrimental ROS production. Next to *SIGLEC3/CD33*, *SIGLEC11* polymorphisms have also been linked to an increased risk of developing AD (88). In this study, AD/dementia-associated genes were categorized according to their relevance to the disease process, and *SIGLEC11* emerged as the most relevant microglial gene associated with AD (88). However, Supplementary Data from this study also revealed that the *SIGLEC16* gene locus is associated with an increased risk of developing AD/dementia, although it remains unclear whether the intact activatory ITAM-signaling minor *SIGLEC16* polymorphism or the non-functional major *SIGLEC16P* pseudogene is involved in AD (88). SIGLEC-14, another activatory ITAM-signaling receptor, has also been suggested to play a role in AD development (102). A deletion polymorphism of *SIGLEC14* has been reported previously (119) and is associated with increased expression of SIGLEC-5, an ITIM-signaling receptor. While SIGLEC-14 is likely expressed in human microglia, as indicated by RNA-seq data (see Figure 3), it remains unclear whether this polymorphism also leads to the expression of SIGLEC-5 in microglia. Altered expression levels of the paired SIGLEC-5 and -14 receptors in patients with the deletion polymorphism of *SIGLEC14* might decrease the capacity of microglia to remove amyloid plaque through phagocytosis, potentially increasing the susceptibility of developing AD (102). Recently, SIGLEC-8 was found to be expressed on microglia (66, 120). Thereby, microglia of aged individuals and patients with late-onset AD expressed higher levels of SIGLEC-8 (66). The functional paralog of SIGLEC-8 in mice, Siglec-F, was upregulated in a subset of microglia at an early stage of disease progression in three mouse models of neurodegeneration. Both SIGLEC-8 and Siglec-F were upregulated in response to IFN-γ treatment in human stem cell-derived microglia models and BV-2 cells, respectively. Overexpression of Siglec-F, SIGLEC-3/CD33, SIGLEC-5, and SIGLEC-8 in BV-2 cells triggered pyroptotic cell death via an inflammasome-involving pathway (66). It remains debatable whether increased expression of SIGLEC-8 in AD patients and Siglec-F in mouse models of neurodegeneration is beneficial or detrimental and whether it also results in pyroptotic microglial cell death in humans. On one hand, decreased reactive microglia might slow down disease progression in later phases, while on the other hand, activated microglia are necessary at earlier stages to clear debris and toxic aggregates.

**Table 1:** Correlation of gene loci, molecules, and enzymes to neuropathological processes.

Neuronal disease phenotype	Disease	Related gene/molecule/enzyme
Synaptopathy and neurodegeneration	Alzheimer’s disease	SIGLEC-3/CD33 full-length receptor CD33M (increased risk) (83, 85, 86)
		SIGLEC-3/CD33 variant receptor CD33m (reduced risk) (83, 85, 86)
		*SIGLEC11*/*SIGLEC16* gene loci (88)
		*SIGLEC14*/*SIGLEC5* gene loci (102)
		Soluble SIGLEC-2/CD22 plasma levels (103)
Synaptopathy	Schizophrenia, autism spectrum disorders, and bipolar disorders	Sialyltransferase ST8SIA2 variants/loss of functions (104–106)
		Decreased sialyltransferase *ST8SIA2* gene expression levels (107)
		Soluble variant NCAM brain/cerebral spinal fluid levels (108)
		Soluble polysialic acid serum levels (109)
		*SIGLEC4*/MAG polymorphism (110, 111)

Although SIGLEC-2/CD22 is primarily expressed on B cells, it has also been found on aged and damage-associated microglia in mice. A study demonstrated that SIGLEC-2 was expressed on microglia and downregulated microglial phagocytic capacity in aged mice (121). However, in a follow-up study in the human brain, CD22 was not found on microglia, but on oligodendrocytes. Here, CD22 was shed from oligodendrocyte as a soluble CD22 (sCD22), binding to sialylated insulin-like growth factor 2 receptor (IGF2R) on microglia, thereby impairing lysosomal trafficking (122). In addition, plasma sCD22 levels of patients negatively correlated with amyloid-β_42_ levels in the cerebrospinal fluid (CSF), but positively correlated with phosphorylated TAU protein levels in the CSF and amyloid-β burden in the brain. Higher plasma sCD22 levels have also been associated with overall decreased cognitive function and faster cognitive decline suggesting a yet unidentified involvement of sCD22 in AD pathogenesis (103). Moreover, sialylation on plaque-associated microglia is increased in 5xFAD mice, potentially leading to decreased microglial plaque-clearing activities mediated through *cis*-interaction with microglial SIGLEC receptors (123). Such hypersialylation is not limited to microglia but appears to be a more widespread phenomenon in AD. AD-associated neurofibrillary tangles (NFTs) and granulovacuolar degenerations (GVDs) have also been shown to exhibit hypersialylation in AD hippocampi (124). To date, it remains unclear whether this hypersialylation in AD results from increased production, insufficient clearance, or failed lysosomal digestion (e.g., by accumulation of poorly cleavable α2,8 Neu5Gc). Next to AD, SIGLECs and sialylation have also been associated with other neurological diseases including Huntington’s disease, frontotemporal dementia and Niemann-Pick disease type C (NPC) (69). In the latter, CD22 was found to be upregulated in *Npc1*-deficient microglia and soluble CD22 was increased in the CSF of Niemann-Pick disease type C patients (125), ultimately resulting in decreased lysosomal trafficking via insulin-like growth factor 2 receptor (122). In frontotemporal dementia, a polymorphism in exon 2 of *CD33* (rs2455069-A>G) has been weakly associated to dementia (126). Furthermore, the glycome including the sialic acid-containing glycans demonstrated remarkable alterations in the brains of Huntington’s disease patients and its mouse models (127, 128).

Overall, there is compelling evidence supporting that a dysregulated ITIM/ITAM signaling axis, arising from polymorphisms in ITIM/ITAM-signaling SIGLECs or ITAM-signaling TREM2, results in an impaired microglial homeostatic function, potentially serving as an underlying mechanism that contributes to the onset and progression of AD and other types of dementias (129).

### 2.5 Involvement of sialylation and complement in psychiatric diseases

Many prevalent neurodegenerative disorders and psychiatric diseases share commonalities in their genetic and molecular pathophysiology (130). Genome-wide association studies (GWAS) have identified genetic variants of enzymes involved in sialic acid biology in several psychiatric diseases. The polysialyltransferase ST8 alpha-N-acetyl-neuraminide alpha-2,8-sialyltransferase 2 (ST8SIA2) contributes to polysialic acid synthesis. Genetic variants of the *ST8SIA2* gene or loss-of-function mutations affecting ST8SIA2 have been shown to be associated with schizophrenia (104–106), bipolar disorder (104), and autism (131, 132) (see Table 1). Additionally, histological analyses have supported the involvement of polysialic acid in these diseases. The expression of polysialylated NCAM is reduced in patients with schizophrenia (133–135). Of note, abnormal concentrations of various NCAM isoforms, including NCAM 105–115 kDa (cN-CAM), NCAM variable alternative spliced exon (VASE), and NCAM secreted exon (SEC) have also been associated with bipolar disorders and schizophrenia (108). Mice with ST8SIA2 deficiency exhibited schizophrenia-like behavioral abnormalities, including cognitive dysfunction, deficits in prepulse inhibition, and increased sensitivity to amphetamine-induced locomotion (136). In children with autism spectrum disorder, *ST8SIA2* gene expression levels were decreased compared to age- and sex-matched controls. Thereby, *ST8SIA2* gene expression levels negatively correlated with the childhood autism rating scale (CARS) score, indicating more serious stereotype behaviors and sensory abnormalities with decreasing *ST8SIA2* expression (107). Furthermore, a correlation between polysialic acid serum levels and structural brain changes related to the schizophrenia spectrum and bipolar disorder was observed, suggesting that soluble polysialic acid, released as part of the disease process in the brain, can be detected in the patients’ serum (109). While no direct link between schizophrenia and the CD33-related SIGLECs has been described so far, patient-specific polymorphisms in the *SIGLEC4/MAG* gene expressed in oligodendrocytes have been significantly associated with the disease (110, 111). However, polymorphisms of complement factor 4, a component functionally closely linked to sialylation, have been associated with schizophrenia (137). As described before, the sialylated glycocalyx is recognized by complement modulators and strongly influences the complement cascade (Figure 1).

Overall, these findings highlight a functional impairment of polysialic acid and the downstream-regulated complement factor 4 as contributing components in the development of schizophrenia and bipolar disorders (106) (Table 1), which share common pathways with neurodegenerative diseases and are associated with an increased risk to develop dementia (130).

### 2.6 SIGLECs can be targeted by antibodies and polysialylated ligands in neurodegenerative diseases

Dysfunction in sialylation or microglial SIGLEC receptor signaling have been associated with neurodegeneration and synaptopathies such as schizophrenia. The microglial response must be precise in both space and time. Thus, microglia should only become activated when and where it is necessary, returning promptly to a homeostatic state once their job is done. Therefore, the inhibitory signaling of SIGLEC receptors on microglia should be conditional. It should maintain microglia in a non-inflammatory, homeostatic state within healthy tissues, yet allow a strong and localized immune or phagocytic response to fight against microbial pathogens or clear debris and pathological aggregates. Nature appears to harness the sialylation pattern on the glycocalyx to maintain a consistently low and homeostatic activation level with a high conditional response. Accordingly, sialylated ligands, which can be removed by endogenous neuraminidases under pathological conditions, might be more suitable for therapeutic approaches compared to the continuous inhibitory activity of an agonistic SIGLEC receptor-specific antibody. However, the development of therapies based on natural sialylated ligands for SIGLECs is still in its infancy due to several methodological limits (see Box 1).

BOX 1 **Challenges for SIGLEC-targeted drug development.**

Currently, the development of drugs targeting SIGLECs faces various challenges. Natural ligands of SIGLECs are often still unknown and chemical synthesis of complex carbohydrates at a large scale is still impossible. Furthermore, innovative and specific tools are needed to screen biologics or molecules targeting human SIGLEC receptors. To this end, several methods were developed to identify the physiological ligands of SIGLEC receptors based on flow cytometry (138), arrays, such as multivalent genetically-encoded liquid glycan arrays (LiGA) of complex N-glycans (139), or glycolipid analysis by multiplexed capillary gel electrophoresis coupled to laser-induced fluorescence detection (xCGE-LIF) (140). The interaction of SIGLEC receptors with their physiological ligands is a low-affinity protein-carbohydrate interaction requiring multivalency between several ligands and clustered SIGLEC receptors for effective binding and signaling. Thus, carbohydrate ligands have to be linked together by a vesicle-like structure or a backbone, for example by binding to the tissue matrix. Thereby, the type of the backbone substantially affects the interaction of the carbohydrate with the SIGLEC receptor (141). Furthermore, monitoring inhibitory SIGLEC signaling in cells is challenging, given that it starts with a short Src kinase-mediated tyrosine phosphorylation activation phase, and promptly succeeded by its inactivation through phosphatases. To overcome this challenge, the ITIM domain of SIGLEC receptors can be switched to an activatory ITAM domain in reporter cell lines to facilitate the monitoring of SIGLEC receptor signaling (142). SIGLEC receptors exhibit considerable diversity between humans and mice, displaying low sequence homology. Thus, human model systems, such as microglia derived from induced pluripotent stem cells, are needed to study SIGLEC functionality, as exemplified in the context of the interaction between TREM2 and SIGLEC-3/CD33 signaling (142). Finally, drug testing should be conducted on humanized SIGLEC transgenic mice models. Several models have now been developed for this purpose (143) and are already employed in the field of ophthalmology to evaluate novel treatment options involving soluble matrix-interacting polysialic acid (144) and polyglycolic/polylactic-conjugated polysialic acid (145).

To date, several antibodies targeting SIGLECs have been tested in cancer, including SIGLEC-2, SIGLEC-3, and SIGLEC-15 (146). In these approaches, the expression of selected SIGLECs on the surface of the malignant cells was leveraged, utilizing SIGLEC-targeting antibodies coupled with cytotoxic agents to effectively deplete the cancerous cells. However, the idea of targeting SIGLECs has also been explored in the context of neurodegenerative diseases. For instance, liposomes coated with Neu5Acα2–6Galβ1-4Glc 1, a sialylated oligosaccharide that binds to SIGLEC-3/CD33, was shown to reduce cell surface expression of SIGLEC-3 by internalization. This approach led to increased phagocytosis by microglia in transgenic mice expressing human SIGLEC-3 (147). Consequently, the concept of inhibiting the suppressive activity of SIGLEC-3 in the brains of AD patients to enhance microglial-mediated clearance of amyloid-β plaques was tested in preclinical and clinical settings. Anti-CD33 antibody lintuzumab was shown to specifically bind full-length SIGLEC-3 (CD33M) and successfully reduced its surface expression (148). As mentioned before, full-length SIGLEC-3 expression has been associated with increased amyloid-β plaque burden in AD (67, 116), but the development of a therapy approach using this antibody was not followed up. Another full-length SIGLEC-3-targeting antibody (AL003), which was developed by Alector Inc., was claimed to block its function and thereby increase the amyloid-β clearance activity of microglia. A phase 1 clinical trial using this antibody started in 2019 and was completed in 2021 in healthy volunteers and AD patients (149). AL003 was generally safe and well tolerated (150). However, to date, this approach has not been further developed.

Although the conditions for the expression of SIGLEC-2/CD22 on microglia in mice is still a matter of debate, the inhibition of SIGLEC-2 with a blocking antibody or its genetic ablation resulted in increased phagocytosis of amyloid-β oligomers, myelin debris, and α-synuclein fibrils. Long-term SIGLEC-2 blockade restored microglial homeostasis and ultimately improved the cognitive function in aged mice (121). Furthermore, SIGLEC-2 blockage restored the age-related decline in microglial surveillance in mice (151). However, the function of SIGLEC-2/CD22 in the human brain is unclear, since SIGLEC-2/CD22 was found to be expressed on human oligodendrocytes and not on microglia (117). Thus, no clinical trial to target SIGLEC-2/CD22 for the treatment of Alzheimer’s disease has been performed so far.

Next, to antibodies, the utilization of polysialic acid has been tested as a therapeutic approach to target SIGLEC receptors in several model systems. Here, the therapy approaches focused on the retina, a part of the central nervous system, which is also affected by age-related inflammatory neurodegeneration. Intravitreal administration of soluble polysialic acid with an average degree of polymerization 20 (polySia avDP20) resulted in a reduction in the reactivity of mononuclear phagocytes, decreased vascular leakage, and prevented complement activation in humanized SIGLEC-11 transgenic mice subjected to laser-induced retinal damage (144). Moreover, polysialic acid linked to a poly(lactic-co-glycolic acid; PLGA)-poly(ethylene glycol; PEG) backbone to create a nanoparticle-like structure was used to prevent damage in an animal model of bright light-induced retinal degeneration (145). This promising avenue involving polysialic acids as therapy for a degenerative retinal disease has now progressed into clinical application (152). Specifically, intravitreal application of oligo- and polysialic acid bound to a PLGA-PEG backbone is currently undergoing phase 2 trials as a treatment for geographic atrophy (152). Although such polymers have been considered to be biocompatible, inflammatory side effects of PLGA/PEG have been described that might interfere with a long-term application in the eye (153).

Systemic application of soluble polysialic acid has also been tested in an inflammatory neurodegenerative disease model. In experimental settings, the systemic administration of soluble polySia avDP20 has been explored in an LPS-triggered animal model of Parkinson’s disease using humanized SIGLEC-11 transgenic mice. Repetitive intraperitoneal administration of polySia avDP20 reduced microglial immunoreactivity and prevented the loss of dopaminergic neurons in the *substantia nigra* (154). Furthermore, intranasally applied soluble polysialic acid with a degree of polymerization 12 (polySia DP12) restored the synaptic activity in the prefrontal cortex of mice deficient in a polysialic acid-producing enzyme. Interestingly, the treatment with polySia DP12 also ameliorated the impaired cognitive performance observed in the polysialic acid-producing enzyme-deficient mice and in two animal models of Alzheimer’s disease (155). Moreover, in a study conducted with double transgenic AD (2 × Tg-AD) mice, sialic acid was added to the mouse chow as a form of treatment (156). The sialic acid-rich diet mitigated cognitive impairment and alleviated symptoms of depression and anxiety. Additionally, it led to a reduction of amyloid-β and neurofibrillary tangle levels while preventing neuronal loss in the brain. Cognitive performance notably improved as demonstrated by the results of the Morris water maze and open field tests. Furthermore, sialic acid inhibited tau hyperphosphorylation and displayed potential to lower blood lipids, thereby possibly preventing vascular diseases (156).

Thus, the current approaches to target sialylation and SIGLECs are mainly performed in animal models and only a few strategies have progressed in clinical trials. Oligo- and polymers of sialic acids might emerge as a promising and innovative therapeutic strategy with potential protective effects against inflammatory neurodegeneration.

## Author contributions

JW: Investigation, Visualization, Writing – original draft, Writing – original draft. TA: Writing – review & editing. GC-R: Writing – review & editing. HL: Writing – original draft, Writing – review & editing. HN: Writing – original draft, Writing – review & editing.
